# CAV2 Regulates Mir-4723/Wnt7A Signalling Axis through Endocytosis and Epithelial-Mesenchymal Transition to Promote Proliferation, Invasion, and Metastasis of Pancreatic Cancer Cells

**DOI:** 10.7150/jca.69617

**Published:** 2022-04-04

**Authors:** Dan Li, Yuting Guo, She Tian, Changhao Zhu, Chengyi Sun

**Affiliations:** 1 Key Laboratory of Hepatobiliary and Pancreatic Surgery and Guizhou Medical University, Guiyang, China.; 2 Department of Hepatic-Biliary-Pancreatic Surgery, The Affiliated Hospital of Guizhou Medical University, Guiyang, China.

**Keywords:** Caveolin-2, MiR-4723, Pancreatic cancer, Endocytosis, Wnt/β-catenin pathway, WNT7A

## Abstract

**Background:** Pancreatic cancer is one of the most aggressive malignancies globally, with no improvement in the cure rates yet.Caveolin-2 (CAV2) has been repeatedly reported to play an important role in cellular transport and signalling and in exhibiting a pro-oncogenic response in a variety of tumours, although its specific action mechanisms in pancreatic cancer are not well documented. MiRNA is recognized as a therapeutic target for a variety of tumours, making it an important regulator of the Wnt/β-catenin signalling pathway. MiR-4723/Wnt7A constitutes an oncogenic signalling axis in pancreatic cancer by targeting and inhibiting Wnt7A through the activation of MiR4723, but its molecular action mechanism remains unexplored. Therefore, in the present study, we investigated the effect of CAV2 on the MiR-4723/Wnt7A pathway and its action mechanism.

**Methods:** We employed TCGA, the GEO database for bioinformatics analysis, cell proliferation assay, wound healing assay, Transwell assay, colony-forming assay, qRT-PCR, and Western blotting to validate the cancer-promoting role of CAV2 in pancreatic cancer and to determine its potential target WNT7A. We then explored CAV2 as a positive regulator of the Wnt7A/β-catenin pathway through immunofluorescence assay, qRT-PCR, and Western blotting. Database analyses, CCK-8 and qRT-PCR revealed that MiR-4723 is an oncogene in pancreatic cancer. Luciferase assay and qRT-PCR revealed that MiR-4723 is a negative regulator of the Wnt7A/β-catenin pathway. To investigate the mechanism of CAV2 action on MiR-4723/Wnt7A, we detected the gene expression of CAV2 through qRT-PCR after MiR-4723 overexpression. Several genes related to endocytosis and epithelial-mesenchymal transition (EMT) were subsequently analysed through immunofluorescence, Western blotting, and qRT-PCR.

**Results:** Overexpression of CAV2 promotes invasion, migration, cloning and metastasis of pancreatic cancer cells. Overexpression of MiR-4723 inhibits CAV2 expression. Here, we are the first to demonstrate that CAV2 exerts a pro-carcinogenic effect on pancreatic cancer through the activation of the Wnt7A/β-catenin signalling pathway.

**Conclusion:** CAV2 can regulate the MiR-4723/Wnt7A signalling axis in pancreatic cancer cell lines by inhibiting endocytosis and promoting EMT, thereby fulfilling the mechanism pro-carcinogenic effects.

## Background

Pancreatic ductal adenocarcinoma (PDAC) is one of the most aggressive cancers known in the world, and its low cure rate and highly malignant nature are the major factors influencing the associated mortality.

Caveolin-2 (CAV2) is a member of the Caveolin family that plays an essential role in intracellular cellular transport and signalling. The high expression of CAV2 has been associated with the progression of several types of tumours, including those of the lung, prostate, kidney, and breast. For example, CAV2 can promote renal cell carcinoma progression by influencing the EGFR/PI3K/Akt pathway 1 and CAV2 is associated with basal-like breast carcinoma and its poor prognosis 2. The literature report suggests that CAV2 regulates various cancer cells' growth and biological activities 3.Further studies have shown that overexpressed CAV2 mechanically promotes PDAC progression and metastasis by disrupting focal adhesion (CCND1, IGTA1 and ZYX) and extracellular matrix (PLOD2, CAST and ITGA1) genes 4.Meanwhile, BRD4 can bind to the CAV2 promoter region and up-regulate the expression of CAV2, thereby promoting PDAC^5^.CAV2 has also been shown to be a target of Mir-29a, and high CAV2 expression is responsible for poor prognosis^6^.

The Wnt/β-catenin signalling plays a vital role in the development and maintenance of homeostasis in several body tissues, and this pathway controls the maintenance of stem cells in numerous tissues and organs. It is involved in pancreatic cancer development by regulating the cell cycle progression, apoptosis, EMT, angiogenesis, stem cells, and the tumour immune microenvironment 7.

MiRNA is a small non-coding RNA that regulates the expression of many genes, many of which are involved in the development of cancer growth. Dysregulated MiRNA expression has been associated with the development of different types of malignancies, especially pancreatic cancer. It has been partially documented that several MiRNAs significantly affect the growth of pancreatic cancer tumours by regulating the Wnt/β-catenin pathway. For example, MiR-455-3p inhibits the Wnt/β-catenin signalling in pancreatic cancer by targeting TAZ, thus acting as an oncogenic agent 8. MiR-774 targets a negative regulator of the Wnt/β-catenin pathway and promotes the stem cell phenotype of pancreatic cancer cells *in vitro*, increasing their tumorigenicity 9. MiR-331-3p can activate the Wnt/β-catenin signalling by targeting ST7L, which affects the growth and drug resistance of pancreatic cancer 10. Moreover, it has been demonstrated that MiR-4723/Wnt7A constitutes a signalling axis that targets and inhibits Wnt7a through the activation of MiR-4723, thereby exerting a significant oncogenic effect in the development and progression of pancreatic cancer, although its mechanism remains unexplored 11.

Endocytosis is an important biological signalling pathway through which cells acquire macromolecules and particulate matter from the extracellular compartment. Recent studies have shown that hypoxia-mediated endocytosis regulates membrane-associated proteins, such as receptor tyrosine kinases (RTK), which is a crucial tumorigenesis mechanism, probably due to the sustained activation of kinase signalling due to ubiquitinated evasion of RTK-like receptors after endocytosis 12, 13. On the other hand, endocytosis can also affect intercellular junctions and cell polarity changes, which may be closely related to the formation of the 'EMT' mechanism and its pro-tumorigenic properties 13.

In this study, we demonstrated that CAV2 could promote invasion, migration, proliferation, and cloning of pancreatic cancer cell lines. We also demonstrated, for the first time, that CAV2 can regulate the MiR-4723/Wnt7A signalling axis in pancreatic cancer cells by inhibiting endocytosis and promoting EMT, thereby accomplishing its pro-cancer effects.

## Materials and Methods

### Cell lines and pancreatic cancer samples

The human PDAC cells and Human pancreatic cells were purchased from the American Type Culture Collection (ATCC). All cell lines were previously identified by ATCC's short tandem repeat (STR) typing. The cells were collected and cultured in 1640, and DMEM media (GIBCO, USA) supplemented with 10% foetal bovine serum (Biological Industries Israel) and incubated at 37 °C under a 5% CO2 atmosphere. This study was approved by the Ethics Committee of the Affiliated Hospital of GuiZhou Medical University, China. Written informed consent was obtained from each participant. The investigation has been conducted in accordance with the Declaration of Helsinki.

### MiRNA overexpression transfection

Before transfection, cells (3 x 10^5^) were inoculated into a 24-well culture plate containing the complete medium at 50% cell density. MiR-4723 mimics or its negative control (RIBOBIO, China) was used for MiRNA transfection according to the reagent manufacturer's instructions. For the transfection reaction, the plate was incubated under a 5% CO_2_ incubator at 37 °C for 72 h.

### Gene overexpression lentivirus infection

Cells (3 x 10^4^) were inoculated into a 6-well-plate containing the complete medium and incubated at 37°C for 12 h until the cell density reached 30%. The HiTransG P infection solution was prepared, and an appropriate virus mix was added to the solution according to the cell MOI and virus titre. The culture medium can be changed midway to ensure cell activity. The efficiency of cell infection was monitored under a fluorescence microscope after 72 h of infection. The lentivirus and its empty vector control were purchased from Genesys (GENE, China).

### Protein extraction and Western blotting

The experimental cells were added to the protein lysate and sonicated in an ice bath for 30 s at a 15-s interval, and the entire step was repeated thrice. After sufficient lysis, the lysate was collected, and the supernatant was centrifuged at 12000 rpm for 10 min to obtain the total protein solution of the cells. A protein standard curve was then prepared by using the BCA method for protein quantification. The total quantified protein was added to the loading buffer, boiled at 100 ℃ for 5 min, and then stored in -20 °C refrigerator. The treated protein was subjected to 10% SDS polyacrylamide gel electrophoresis and then transferred onto polyvinylidene fluoride (PVDF) membrane. The membrane was treated with 5% skimmed milk and washed 5 times, followed by treatment with diluted primary antibody added as required and incubated overnight for (>10 h) at 4 °C, washed 5 times, and then incubated with peroxidase-conjugated goat anti-rabbit IgG antibody (1:2000 diluted), washed, and then added to the developer for exposure analyses. The primary antibodies were purchased from Abcam (Cambridge, UK), while the secondary antibodies were purchased from Proteintech Group, Inc. (Chicago, USA). All antibodies were diluted to 1:1000 unless otherwise stated.

### qRT-PCR analysis

Total RNA was extracted from the treated PAN-1 and BXPC-3 cells with the TRIzol reagent (Invitrogen), then determined the RNA concentration. Complementary DNA (cDNA) synthesis was performed using a reverse transcription kit (Invitrogen), and PCR was performed using the SYBR Green Premix Ex Taq (Takara, Japan) on the Applied Biosystems 7900 (Applied Biosystems). The qRT-PCR was performed under the following parameters: 50 °C for 2 min, 95 °C for 2 min, 40 cycles of 95 °C for 15 s, and a final 60 °C for 1 min. Glyceraldehyde 3-phosphate dehydrogenase (GAPDH) was selected as an internal reference for the target genes. The gene expression was quantified by the 2^- ΔΔCt^ method. All reactions were run in triplicate. The primer sequences used are as follows:

Wnt7A-F: 5'AGAAGCCACTGTCGTACCG3'; Wnt7A-R: 5'CTGGGGAGCCGTCTTGT3'; CAV2-F: 5'CGTGCCTAATGGTTCTGCCT3'; CAV2-R: 5'CGCTCGTACACACAATGGAGCA3'; β-PDGFR-F: 5'CCTCCATCCCTCTGTTCTCC3'; β-PDGFR-R: 5'CTAGCCCAGTGAGGTTGGTC3'; EGFR-F: 5'GCCAAGGCACGAGTAACAAG'; EGFR-R: 5'AGGGCAATGAGGAVATAACCA'; PAR3-F: 5'GGGGACGGCCACATGAAAG'; PAR3-R: 5'TTCCAAGCGATGCACCTGTAT'; Claudin-6-F: 5'TTCATCGGCAACAGCATCGT'; Claudin-6-R: 5'GGTTATAGAAGTCCCGGATGA'; E-Cadherin-F: 5'CGCATTGCCACATACA'; E-Cadherin-R: 5'CGTTAGCCTCGTTCTCA'; Vimentin-F: 5'CGCTTCGCCAACTACAT'; Vimentin-R: 5'AGGGCATCCACTTCACAG'; Snail-F: 5'CGGGATCCTTCTTCTGCGCTACTGCTGCG'; Snail-R: 5'CGGAATTCGGGCAGGTATGGAGAGGAAGA'; β-catenin-F: 5'GCTGGTGACAGGGAAGACATC3'; β-catenin-R: 5'CGAATCAATCCAACAGTAGCCTTTATCA3'; GAPDH-F: 5'-AATCCCATCACCATCTTCC-3'; GAPDH-R: 5'-CATCACGCCACAGTTTCC-3'.

### Cell Counting Kit-8 (CCK-8) assay

Cell Counting Kit-8 (CCK-8; Dojindo Co., Ltd, Kumamoto, Japan) was used to detect the proliferative capacity of cells. After 12-24 hours of transfection, transfected cell suspensions were plated in 96-well plates (100 μL/well; 4000 cells/well) and incubated at 37°C under a 5% CO_2_ atmosphere. During incubations at 0, 12, 24, and 48 h, the CCK-8 solution (10 μL/well) was added to each well, and the cells were incubated for 1-4 h. The absorbance values of the cell suspensions at 450 nm were measured using an enzyme marker. The experiment was repeated thrice.

### Wound healing and colony-forming assay

The wound-healing assay was performed to test the migration ability of the tumour cells. After grouping the various cell types, the cells were cultured in 6-well plates. When the cells were spread to >80% of the well plate, a monolayer of the cells was scraped pensively using a 200-μL pipette tip to make 2 linear regions without cells that were then cultured in a serum-free medium. Separate images were taken at 0 h, 24 h, and 48 h to capture and measure the migration distance between the linear region at different time points. A colony-forming assay was performed to test the clonogenic ability of the tumour cells. Logarithmically grown cells of all types were collected, digested with 0.25% trypsin, and blown several times until they were observed as single cells under the microscope, followed by suspending in a medium containing 10% foetal bovine serum, diluted multiple times, and inoculated in culture dishes; the plate was placed in an incubator for 2 weeks, terminated after cloning was observed with the naked eye and the cells were fixed in methanol for 15 min and the photographs of the stained cells were taken for enumeration.

### Transwell

Approximately 5 x 10^4^ cells were grown in transwell chambers pre-coated with Matrigel (BD Biosciences, San Jose, California) in a total culture volume of 200 μL. Complete medium with 10% FBS was added to the lower chamber and incubated for 24 h at 37°C in a 5% CO_2_ incubator. The incubated upper chamber was removed, washed thrice with PBS, and soaked in 95% ethanol for 10 min, after which the membrane was stained with 0.1% crystal violet for 10 min. After staining, the cells were naturally dried in the air and washed 5 times with PBS. Finally, the cells were counted under an inverted light microscope, and their photographs were captured.

### Statistical analyses

SPSS v13.0 (IBM, Chicago, IL, USA) and the R language software were used for statistical analyses. The databases were derived from the TCGA and GEO databases. Data were presented as the mean ±SD and analysed by Student's two-tailed *t*-test. One-way ANOVA (two-sided) was applied for paired multiple comparisons. *P* < 0.05 was considered to indicate statistical significance.

## Result

### CAV2 expression was upregulated in pancreatic cancer

**Figure 1:**
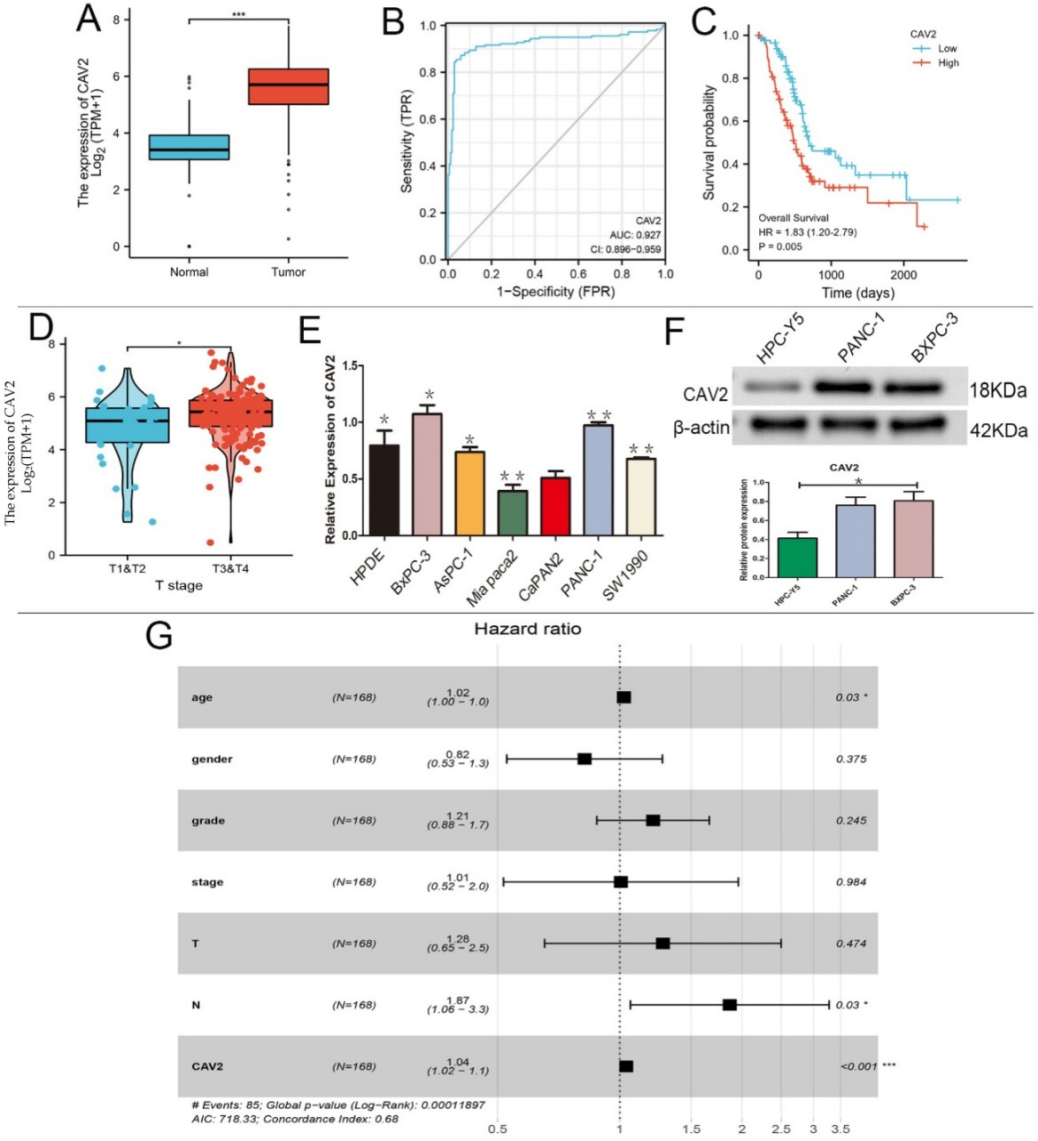
** A.** Differences in the CAV2 expression in normal pancreatic tissues and pancreatic cancer tissues. **B.** ROC curve of the CAV2 expression in pancreatic cancer-specific diagnosis. **C.** Plot of survival rates in pancreatic cancer tissues categorised into high and low CAV2 expression groups with controlled analysis. **D.** Differences in the CAV2 expression in pancreatic cancer at its TNM staging. **E.**qRT- PCR assay, the gene expression of CAV2 in various types of pancreatic cancer cells. **F.** Western blotting,protein expression of CAV2 in human pancreatic cells and in two types of human pancreatic cancer cells and its grayscale value detection histogram. G**.** Plot based on the forest plot of CAV2 expression in 168 pancreatic cancer patients with risk grading. (*P < 0.05; **P < 0.01; ***P < 0.001)

Based on the GEO-DATASET and TCGA databases, we performed bioinformatics analyses. CAV2 was expressed at low levels in normal pancreatic tissues, and high levels in pancreatic cancer tissues (e.g., Fig. 1A), and its expression was found to increase with age in 168 pancreatic cancer patients (e.g., Fig. 1E, P < 0.05). CAV2 had specific diagnostic significance in the area under the pancreatic cancer-specific ROC curve of 0.927 (e.g., Fig. 1B), and the pancreatic cancer group with high CAV2 expression showed worse survival when compared to the low expression group (e.g., Fig. 1C). The CAV2 expression also showed significance in the TNM staging of pancreatic cancer, with a higher expression in stages T3 and 4 stages when compared to that in T1 and T2 (e.g., Fig. 1D); in the regional lymph node, the N stage also showed a positive statistical correlation (e.g., Fig. 1G, *P* < 0.05). By qRT-PCR analysis, we found that CAV2 gene expression was highest in BXPC-3 and PANC-1 in various types of pancreatic cancer cells (e.g., Fig. 1E, *P* < 0.05).We also explored the CAV2 protein expression in human pancreatic cells HPC-Y5 with PANC-1 and BXPC-3 and found that the protein expression was significantly down-regulated in HPC-Y5 (e.g., Fig. 1F, *P* < 0.05).

### CAV2 upregulation promotes proliferation, cloning, invasion, and metastasis of pancreatic cancer cells

**Figure 2:**
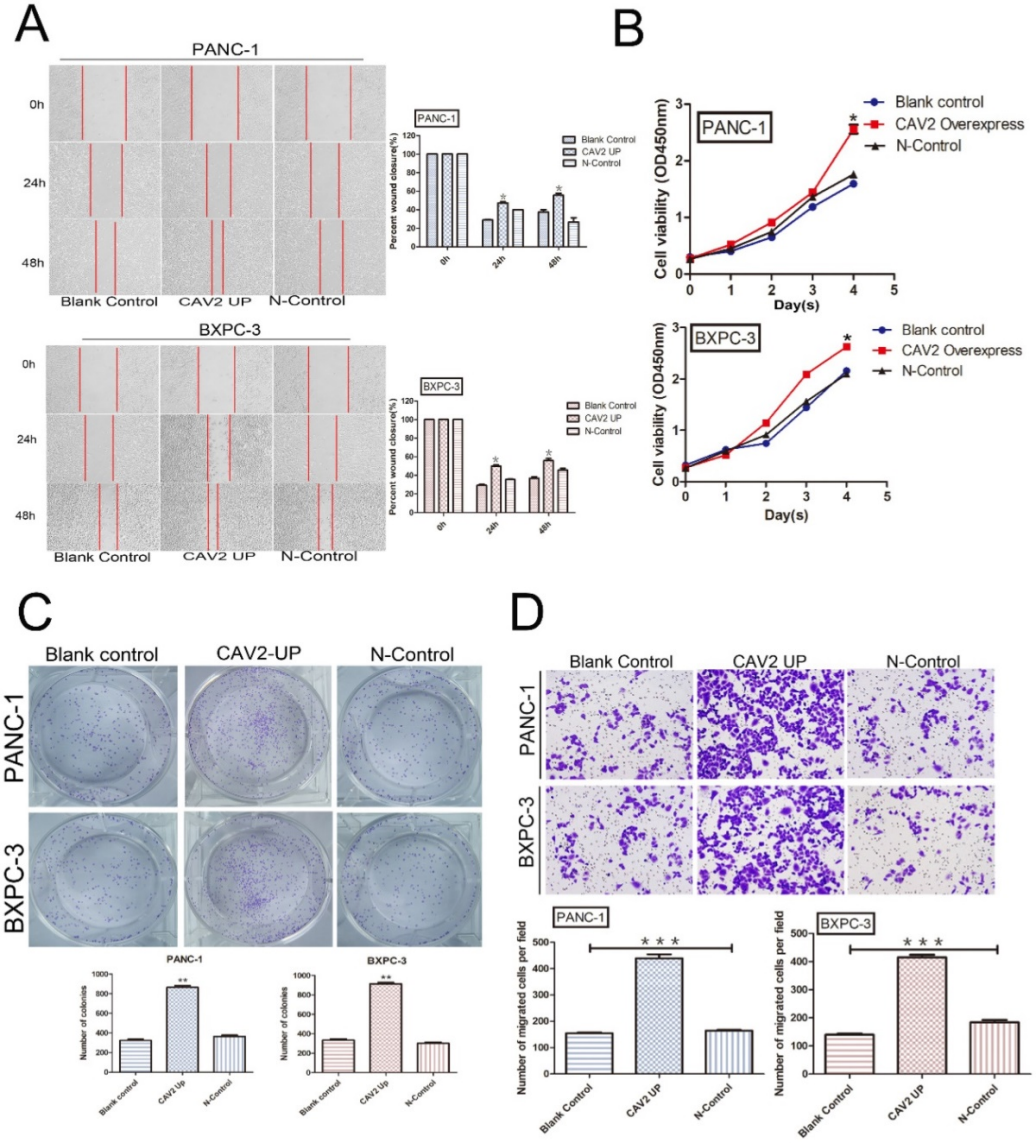
** A.** Wound healing assay, migration, and repairability of pancreatic cancer cells overexpressing CAV2 gene and its control group and its statistical histogram.** B.** CCK-8 assay, absorbance at 450 nm of pancreatic cancer cells overexpressing *CAV2* and its control group to demonstrate their proliferation ability. **C.** Colony-forming assay, cloning ability of pancreatic cancer cells overexpressing CAV2 gene and its control group, and its statistical graph of the number of cloned cells. **D.** Transwell assay, the invasive metastatic ability of pancreatic cancer cells overexpressing *CAV2* and its control group, and its invasive metastatic cell count statistics. (*P < 0.05; **P < 0.01; ***P < 0.001)

We explored the cell biological functions of pancreatic cancer cells PANC-1 and BXPC-3 through lentiviral transfection to upregulate their CAV2 expression. PANC-1 and BXPC-3 were each divided into 3 groups: pancreatic cancer cells without any treatment (blank control); pancreatic cancer cells overexpressing CAV2 via lentiviral infection (CAV2 overexpress); and pancreatic cancer cells after lentiviral infection with the blank vector (N-Control). We found that the pancreatic cancer cells overexpressing CAV2 demonstrated a significant increase in migration, and their repairability was comparable to that of the control group (Fig. 2A). At the same time, the rate of change in their absorbance values at 450 nm also tended to increase when compared to that in the control group, thus representing an increase in their proliferation ability (Fig. 2B). The cloning ability of pancreatic cancer cells overexpressing CAV2 was significantly increased compared to that in the control group (Fig. 2C). In the Transwell assay, we noted that pancreatic cancer cells overexpressing CAV2 showcased a significantly higher ability to invade and metastasize when compared to the control group (Fig. 2D). All of the above data were found to be statistically significant (*P* < 0.05).

### Upregulation of CAV2 targets Wnt7A, thereby activating the Wnt7A/β-catenin pathway and contributing to pancreatic cancer development

**Figure 3:**
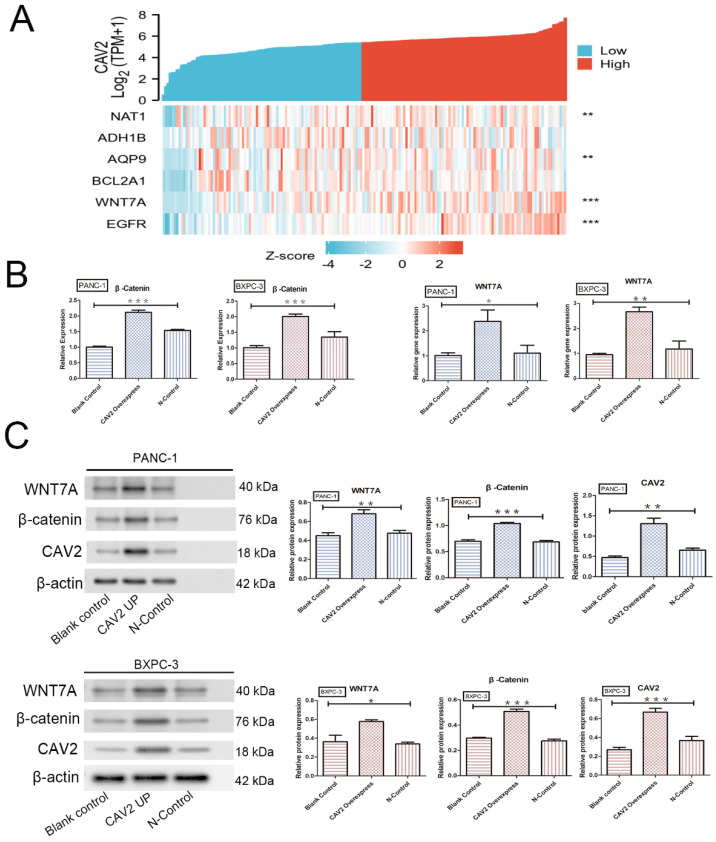
** A.** Heat map of the expression trends between CAV2 and multiple other genes in 182 pancreatic cancer cases and their precancerous tissues;** B.** qRT-PCR, the effect of the overexpression of CAV2 on the Wnt7A and β-catenin gene expression in pancreatic cancer cells and their controls; **C.** Western blotting, the effect of overexpression of CAV2 on Wnt7A and the β-catenin protein expression in pancreatic cancer cells and their controls on Wnt7A and the β-catenin protein expression, and its grayscale value detection histogram. (*P < 0.05; **P < 0.01; ***P < 0.001)

**Figure 4:**
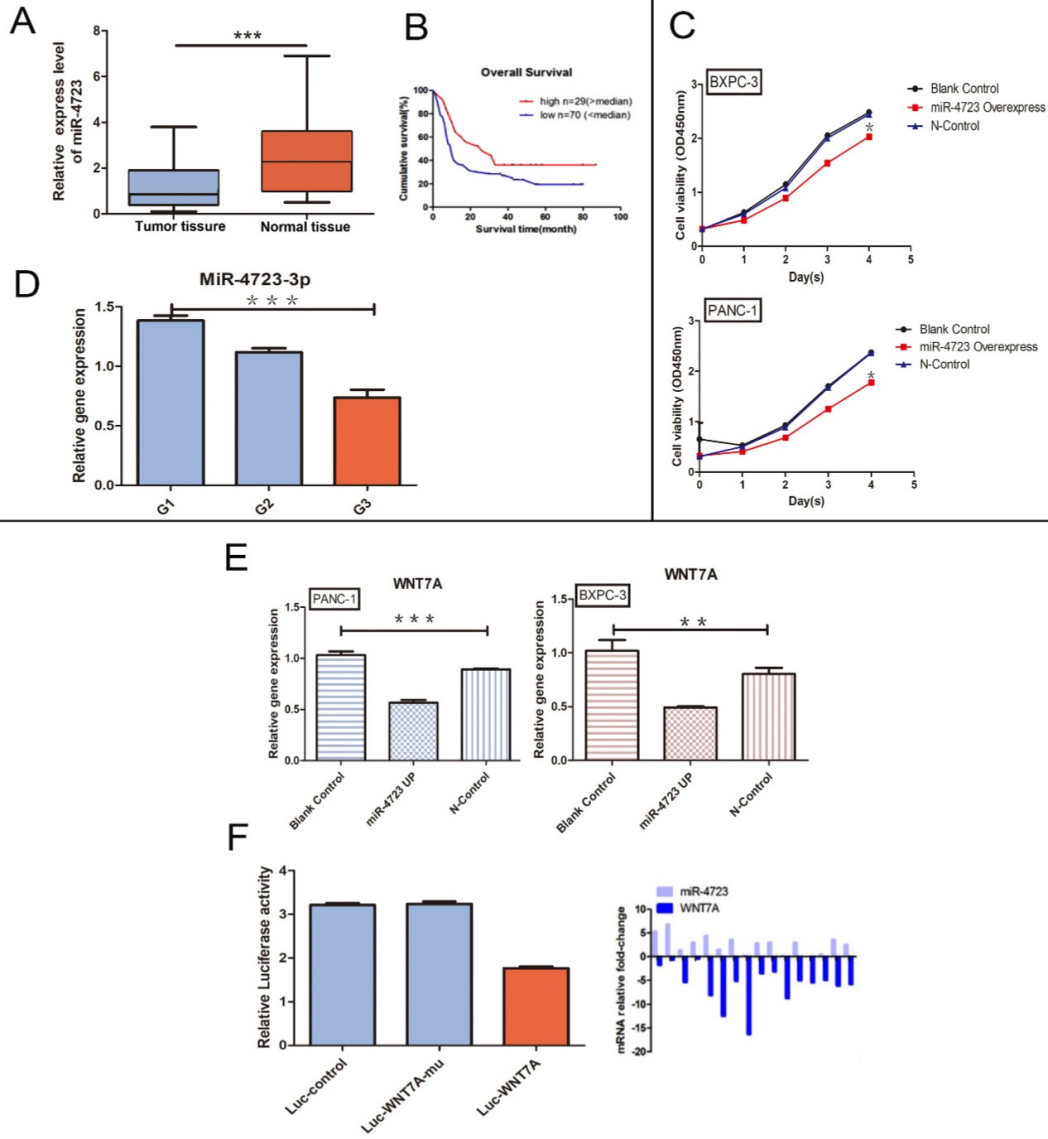
** A.** The differential expression of MiR-4723 in pancreatic cancer and normal pancreatic tissues. **B.** Plots of the survival rates of MiR-4723 in pancreatic cancer tissues categorised into the high and low expression groups, with controlled analysis. **C.** CCK-8 assay, absorbance values at 450 nm in pancreatic cancer cells overexpressing *MiR-4723* gene and their controls. **D.** qRT- PCR assay, the gene expression of MiR-4723 in pancreatic cancer tissues at different stages of differentiation.** E.** qRT-PCR assay, the gene expression of Wnt7A in pancreatic cancer cells and their controls after MiR-4723 overexpression.** F. Luciferase, wild-type Wnt7A luciferase activity after the overexpression of MiR-4723**. (*P < 0.05; **P < 0.01; ***P < 0.001)

The Wnt/β-catenin signalling pathway is essential for embryonic development and adult tissue homeostasis and regeneration 14, while β-catenin is a critical Wnt sensor 15. To investigate the relationship between CAV2 and this pathway, we screened 182 pancreatic cancer-related tumour and paracancer tissue data with reference to the TCGA database and performed Zscore transformations to analyse the expression trends of CAV2 and several related genes. These analyses yielded results showing that the correlation of expression trends between CAV2 and Wnt7A was significantly higher in pancreatic cancer and its paracancer tissues. The expression relationship between the two established a positive correlation (Fig. 3 A, *P* < 0.001). qRT-PCR analyses revealed that the gene expression of Wnt7A and β-catenin was significantly increased in pancreatic cancer cells overexpressing CAV2 (Fig. 3B, *P* < 0.05). Western Blotting results revealed that the protein expression of Wnt7A and β-catenin were significantly increased in pancreatic cancer cells overexpressing CAV2, and their greyscale values were statistically significant (Fig. 3C, *P* < 0.05). Our immunofluorescence assay revealed that the intranuclear expression level of β-catenin was significantly higher in CAV2 overexpressing pancreatic cancer cells when compared to that in the two control groups (Fig. 4A).

### MiR-4723 inhibits pancreatic cancer progression by targeting Wnt7A to regulate the Wnt/β-catenin pathway negatively

Based on the GEO database analyses, MiR-4723 was significantly downregulated in the pancreatic cancer tumour tissues (Fig. 4A), with a higher survival rate in the high MiR-4723 expression group of pancreatic cancer patients when compared to the corresponding low expression group (Fig. 4B). The MiR-4723 expression was examined separately for each of the 3 differentiation levels of pancreatic cancer tissues, showing higher MiR-4723 expression at higher differentiation levels (Fig. 4 D). The overexpression of MiR-4723 in pancreatic cancer cells assayed its proliferative capacity, and we found that its value-added capacity decreased after MiR-4723 upregulation compared with that of the control group (Fig. 4C). To further verify the relationship between MiR-4723 and Wnt7A/β-catenin, we performed qRT-PCR assays and concluded that the Wnt7A expression was downregulated in pancreatic cancer cells after the upregulation of MiR-4723 (Fig. 4E). On the other hand, the luciferase assays revealed that, after the overexpression of MiR-4723 in both pancreatic cancer cell lines, the luciferase activity of wild-type Wnt7A was reduced (Fig. 4F).

### CAV2 upregulation may affect MiR-4723 and activate Wnt/β-catenin through EMT and endocytosis

**Figure 5:**
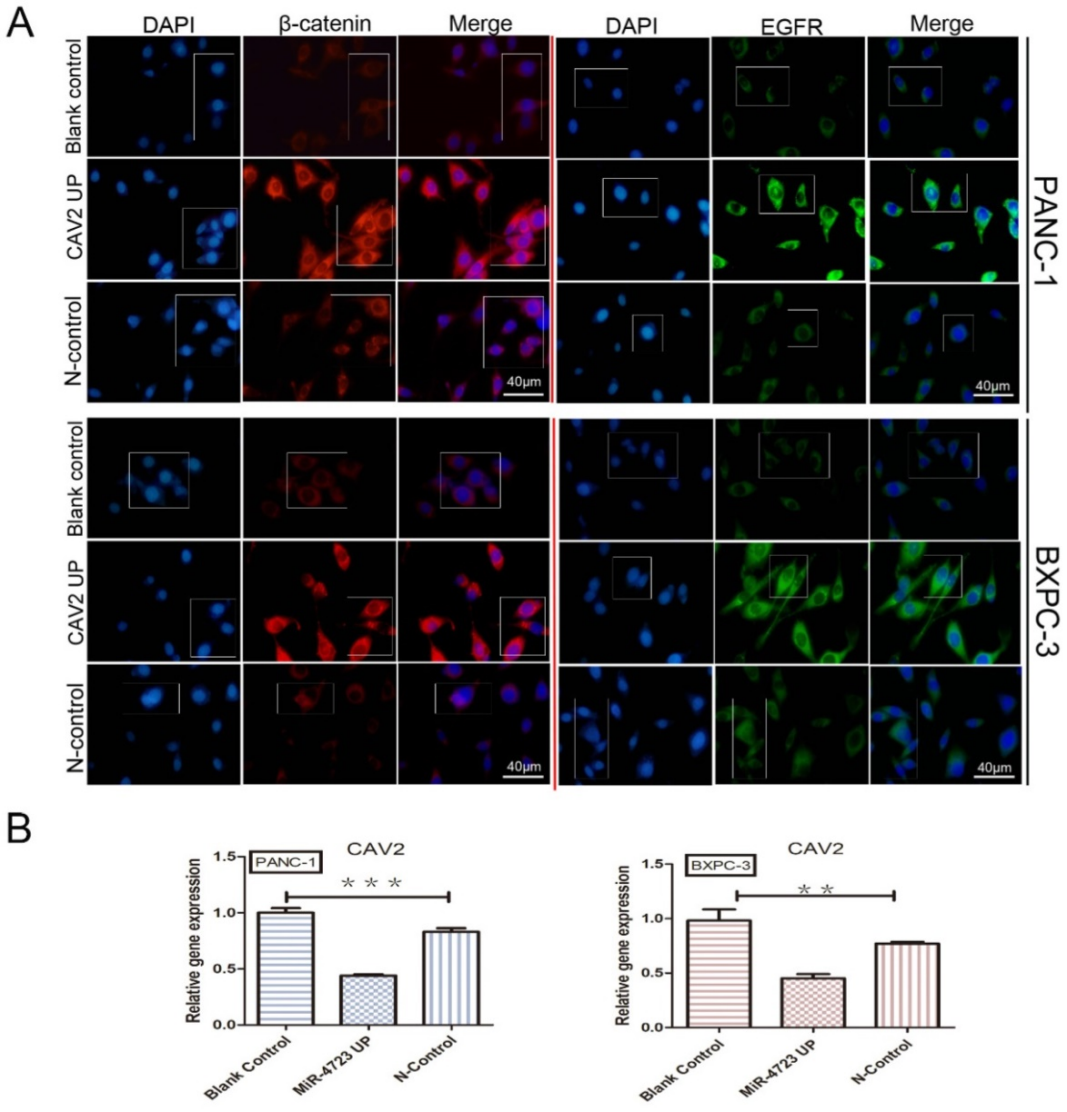
** A.** Immunofluorescence plots, β-catenin, and EGFR intranuclear expression levels in overexpressing CAV2 pancreatic cancer cell lines and their controls. **B.** qRT-PCR, the CAV2 expression levels in overexpressing MiR-4723 pancreatic cancer cell lines and their controls. (*P < 0.05; **P < 0.01; ***P < 0.001)

We first concluded, by qRT-PCR, that the gene expression of CAV2 was significantly downregulated in pancreatic cancer cell lines overexpressing MiR-4723 compared to that in the control cells (Fig. 5B). The correlation between the expression trends of CAV2 and EGFR was significantly higher in pancreatic cancer and its paraneoplastic tissues, and the relationship between the two expressions indicated a positive correlation (Fig. 3A, *P* < 0.001), after which we noted that the intranuclear EGFR expression level was significantly upregulated in CAV2 overexpressing pancreatic cancer cells relative to that in the control group by immunofluorescence assay (Fig. 5A).

In addition, we divided PANC-1 and BXPC-3 into 4 groups each, namely: pancreatic cancer cells without any treatment (blank control); pancreatic cancer cells after MiR-4723 overexpression (MiR-4723 UP/MiR-4723 Overexpress); pancreatic cancer cells overexpressing Wnt7A (Wnt7A UP/ Wnt7A Overexpress); and pancreatic cancer cells overexpressing CAV2 (CAV2 UP/CAV2 Overexpress). To investigate the action mechanism, we selected the following genes: *EGFR* and *PDGFR* 16, 17, which are related to the RTK; E-Cadherin 18, Vimentin 19, and Snail 20, which are connected to the EMT; and PAR3 21, 22, a polarity marker expression gene related to endocytosis, Claudin-6 23, 24.

**Figure 6:**
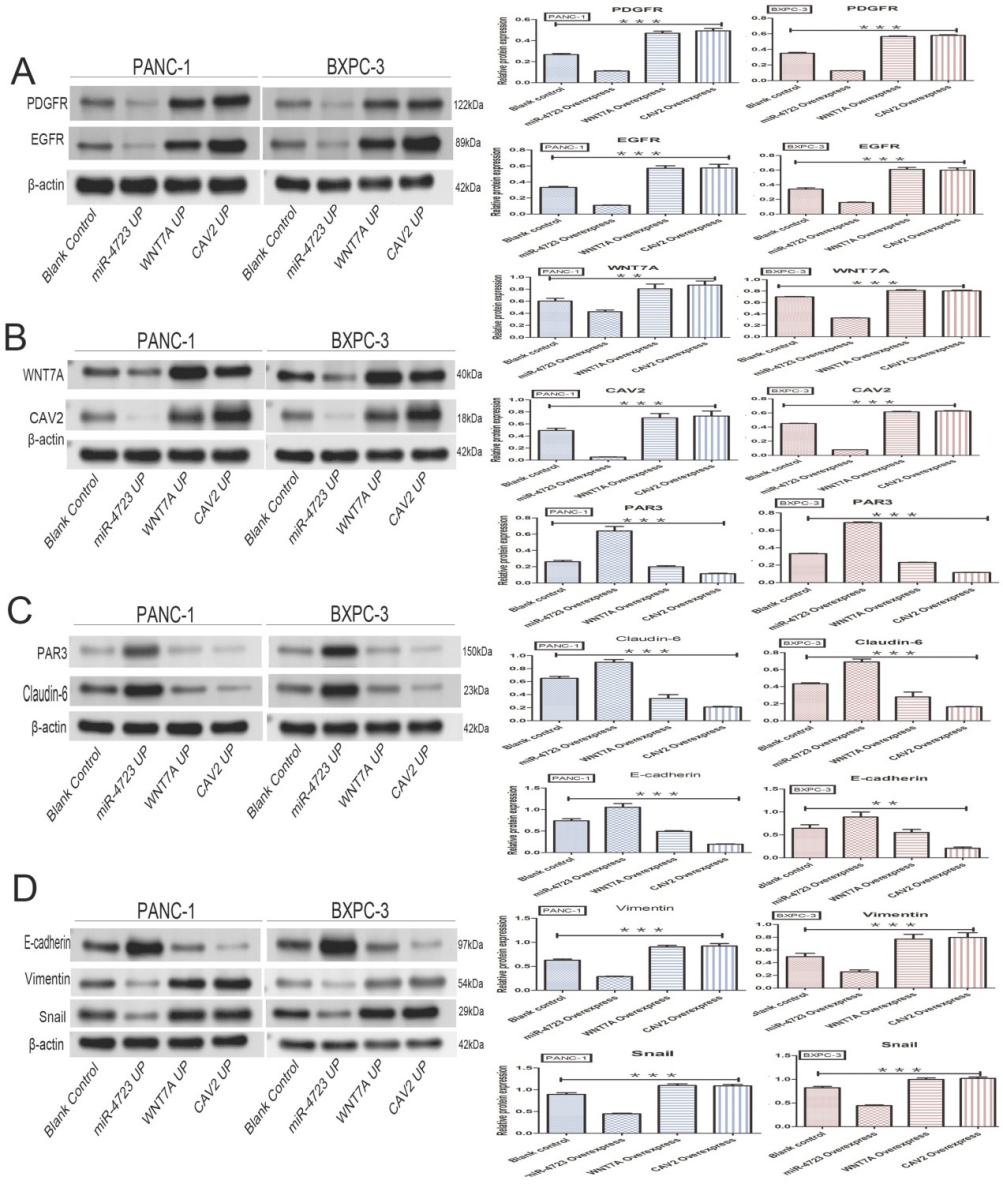
** A.** Western blotting, the effect of overexpression of MiR-4723, Wnt7A, and CAV2 on the PDGFR and EGFR protein expression in pancreatic cancer cells, and its grayscale detection histogram. **B.** Western blotting, the effect of overexpression of MiR-4723, Wnt7A, and CAV2 on the Wnt7A and CAV2 protein expression in pancreatic cancer cells, and its grayscale detection histogram. **C.** Western blotting, the effect of overexpression of MiR-4723, Wnt7A, and CAV2 on the PAR3 and Claudin-6 protein expression in pancreatic cancer cells, and a histogram of their grayscale values. **D.** Western blotting, the effect of overexpression of MiR-4723, Wnt7A, and CAV2 on E-cadherin, Vimentin, and Snail protein expression in pancreatic cancer cells, and their grayscale-detection bar graph. (*P < 0.05; **P < 0.01; ***P < 0.001)

The following results were obtained from the Western blotting analysis. When compared to the control group, the protein and gene expression of PDGFR and EGFR was significantly lower in pancreatic cancer cells overexpressing MiR-4723, while it was significantly higher in cells overexpressing CAV2 or Wnt7A (Fig. 6A,7A). The Wnt7A protein and gene expression was reduced in pancreatic cancer cells overexpressing MiR-4723 and increased in cells overexpressing CAV2. In addition, the CAV2 protein expression was reduced in pancreatic cancer cells overexpressing MiR-4723 and increased in cells overexpressing Wnt7A (Fig. 6B,). The expression of PAR3 protein and gene was significantly increased in pancreatic cancer cells overexpressing MiR-4723 and decreased in cells overexpressing CAV2 or Wnt7A; the Claudin-6 protein expression was increased in pancreatic cancer cells overexpressing MiR-4723 and decreased in cells overexpressing CAV2 or Wnt7A (Fig. 6C,7B). The E-cadherin protein expression was significantly increased in pancreatic cancer cells overexpressing MiR-4723, while it was decreased in cells overexpressing CAV2 or Wnt7A; the Vimentin and Snail protein expression was decreased in pancreatic cancer cells overexpressing MiR-4723 but increased in pancreatic cells overexpressing CAV2 or Wnt7A (Fig. 6D,7C). The grey-scale values were statistically significant in all groups (*P* < 0.05).

## Discussion

Pancreatic cancer is presently one of the most aggressive malignancies known globally, with a 5-year survival rate of only 10%; so far, pancreatic cancer is mainly treated by surgery 25, which has an inferior prognosis. The search for therapeutic approaches and targets for pancreatic cancer has thus become a recognized challenge globally. Increasing research has shown that a variety of MiRNAs play a significant role in pancreatic cancer 26, 27, that MiR-4723 can inhibit the development of pancreatic cancer, and that MiR-4723 targets Wnt7A through the Wnt/β-catenin pathway to inhibit the growth, migration, and invasion of pancreatic cancer 11.

Caveolin, an integral membrane protein found in the outer cell membrane, endoplasmic reticulum, Golgi apparatus, and transit vesicles 28, comprises caveolin proteins, cholesterol, glycosphingolipids, and GPI-anchored proteins. Caveolin exists in 3 different types: Caveolin 1 (CAV1), Caveolin 2 (CAV2), and Caveolin 3 (CAV3), which have highly similar structures and amino acids 29. CAV2 is involved in lipid metabolism, epidermal activity, and human carcinogenesis, thereby playing an essential role in a variety of cancer mechanisms and being associated with lymph node metastasis of tumours 30, 31.

This experiment aimed to explore the effects of CAV2 and MiR-4723 on pancreatic cancer and deeply explore the role and mechanism of CAV2 in MiR-4723/Wnt7A.

First, the use of bioinformatics analysis with reference to the TCGA and GEO databases revealed that the CAV2 expression was significantly upregulated in pancreatic cancer compared to that in normal pancreatic tissues and that the prognosis and survival of patients decreased with the expression of CAV2. The ROC curve also demonstrated that the CAV2 expression was significantly specific in the diagnosis of pancreatic cancer. Second, the results were obtained through different cytological experiments suggesting that CAV2 promotes migration, repair, invasion, cloning, and proliferation of pancreatic cancer cells. In summary, we conclude that CAV2 plays a pro-carcinogenic role in pancreatic cancer and that the upregulation of CAV2 promotes the development and metastasis of pancreatic cancer.

To investigate the mechanism by which CAV2 promotes pancreatic cancer, we identified Wnt7A as a potential target of CAV2 by using the TCGA database and bioinformatics analyses, whereby CAV2 seems to be the key regulator of Wnt7A. Through qRT-PCR and Western blotting, we determined that the upregulation of CAV2 promotes the expression of Wnt7A, β-catenin gene, and proteins in pancreatic cancer cells. In contrast, the intranuclear expression level of β-catenin is upregulated with an increase in CAV2 through immunofluorescence. A literature review revealed that Wnt7A is significantly expressed in various cancers 32-34. Wnt7A has also been reported to be an essential ligand for the activation of Wnt/β-catenin and β-catenin/MMP9 34, 35, making it the only ligand that activates the intact β-catenin/TCF signalling axis 36. It is thus reasonable to conclude that CAV2 can activate the Wnt/β-catenin pathway by targeting Wnt7A and is a positive regulator of the Wnt/β-catenin pathway.

According to the review of published and present research, the expression of MiR-4723 was significantly downregulated in pancreatic cancer, thus serving as a tumour suppressor in pancreatic cancer. We also demonstrated that MiR-4723 could affect the Wnt/β-catenin pathway by inhibiting Wnt7A through qRT-PCR, luciferase assay, and Western blotting. Therefore, we concluded that MiR-4723 is a negative regulator of Wnt/β-catenin that targets Wnt7A. Therefore, we plan to explore the MiR-4723/Wnt7A pathway at a deeper mechanistic level in the future.

Our qRT-PCR results suggest that the *CAV2* expression was downregulated in pancreatic cancer with MiR-4723 upregulation. The results by Western blotting showed that Wnt7A was positively regulated with both the sides of CAV2, while MiR-4723 was negatively regulated with the former two. This observation led to the conclusion that CAV2 and MiR-4723 act as mutual inhibitors in the Wnt7A/β-catenin pathway.

The activation of Wnt/β-catenin is inextricably associated with endocytosis, and its signalling is regulated because of the endocytosis of cell adherence plaques, which in turn control cell invasion and metastasis, the absence of which often leads to the development of tumorigenesis 37. The endocytosis-related proteins PAR3 and Claudin-6, which control cell polarity, are closely associated with tumour polarity, permeability, and adhesion. The knockdown of PAR3 promotes the development of pancreatic cancer, and its functional role may be mediated through an endocytic bridging protein, Numb 22, 38, 39. At the same time, Claudin-6, a member of the claudin family (CLDN), has been partially studied for its reduced expression in gastric cancer 23, and it has also been reported to be expressed in some tumours, but not in peritumoral tissues 24. Our study showed that the upregulation of CAV2 or Wnt7A results in the downregulation of the expression of both endocytosis-related polar proteins, whereas the upregulation of MiR-4723 showed an opposite result, thus leading to the inference that the upregulation of CAV2 inhibits endocytosis to an extent, thereby affecting the MiR-4723/Wnt7A pathway and promoting the development of pancreatic cancer.

Wnt/β-catenin is also closely associated with EMT and may be associated with cancer cells' acquisition of stemness characteristics 40. In contrast, CAV2 induces various epithelium-related molecules, including Vimentin, N-Cadherin, E-Cadherin, MMP13, and MYCL, which play important roles in prostate cancer cell motility 41. miRNAs are also the key regulators of EMT; for example, MiR-200 can inhibit EMT development by targeting p53 42; MiR-34a can inhibit EMT development by targeting SMAD4 43, and MiR-155-5P can inhibit MMT by targeting GSK-3β 44. RTK receptor (e.g., EGFR and PDGFR) and E-cadherin occupy an important position in the EMT process. The latter is an adhesion molecule that plays an important inhibitory role in the invasion and metastasis of almost all epithelial malignancies, and it is usually accompanied by the loss of function, gene inactivation, and hypermethylation inactivation during tumour progression 45. On the other hand, Snail is a strong inhibitor of E-cadherin, and epithelial cells ectopically expressing Snail adopt a fibroblast phenotype to gain tumorigenicity and aggressiveness 46. Our study revealed that the upregulation of CAV2 and Wnt7A caused an upregulation of EGFR, PDGFR, Vimentin, and Snail expression and the downregulation of E-cadherin expression, while the overexpression of MiR-4723 resulted in an opposite outcome relative to that of the former. CAV2 may affect the MiR-4723/Wnt7A pathway through EMT. Thereby CAV2 may affect the MiR-4723/Wnt7A pathway through EMT, thus promoting pancreatic cancer.

In summary, we conclude that CAV2 is an oncogenic gene in pancreatic cancer that exerts its oncogenic effect by targeting Wnt7A to activate the Wnt/β-catenin signalling pathway activity. At the same time, MiR-4723 is a negative regulator of the Wnt7A/β-catenin signalling pathway and possibly a potential target to inhibit the Wnt7A/β-catenin pathway activity. MiR-4723 and CAV2 are mutually suppressive in the Wnt7A/β-catenin pathway, and CAV2 can regulate the MiR-4723/Wnt7A pathway by inhibiting endocytosis promoting EMT, which ultimately promotes the proliferation, invasion, and metastasis of pancreatic cancer.

Thus, unravelling the role of CAV2 and MiR-4723 in pancreatic cancer and further exploring their mechanisms can facilitate the expansion of the knowledge about the molecular mechanisms of pancreatic cancer development to lay a theoretical foundation for the further exploration of early diagnosis, clinical behaviour prediction, immunotherapy, or targeted biological therapy for pancreatic cancer treatment.

## Figures and Tables

**Figure 7:**
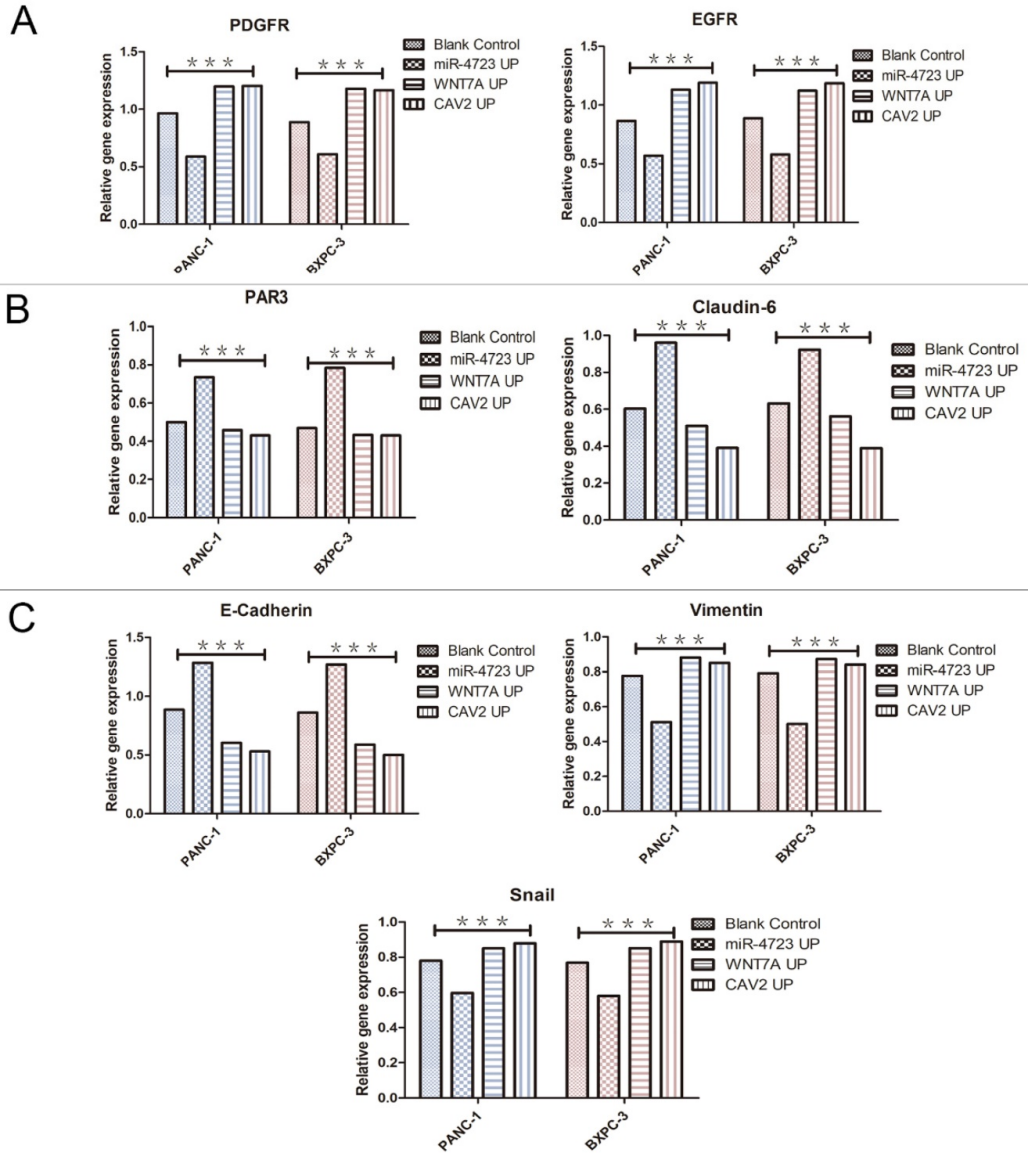
** A.** qRT-PCR, the PDGFR and EGFR gene expression levels on the overexpression of MiR-4723, Wnt7A, and CAV2 and their controls. **B.** qRT-PCR, the PAR3 and Claudin-6 on the overexpression of MiR-4723, Wnt7A, and CAV2 and their controls. **C.** qRT-PCR, the E-cadherin,Vimentin and Snail gene expression levels on the overexpression of MiR-4723, Wnt7A, and CAV2 and their controls. (*P < 0.05; **P < 0.01; ***P < 0.001).
